# Wild-type IDH1 inhibits the tumor growth through degrading HIF-α in renal cell carcinoma

**DOI:** 10.7150/ijbs.54401

**Published:** 2021-03-25

**Authors:** Song Chen, Yejinpeng Wang, Yaoyi Xiong, Tianchen Peng, Mengxin Lu, Lian Zhang, Zhongqiang Guo

**Affiliations:** 1 Department of Urology, Zhongnan Hospital of Wuhan University, Wuhan 430071, China.; 2 Department of Cardiovascular Surgery, Zhongnan Hospital of Wuhan University, Wuhan 430071, China.; 3 Department of Nephrology, Zhongnan Hospital of Wuhan University, Wuhan 430071, China.

**Keywords:** IDH1, renal cell carcinoma, HIF-1α, HIF-2α, α-KG

## Abstract

The purpose of our study was to explore the effect and intrinsic mechanism of wild-type IDH1 and its substrate α-KG on renal cell carcinoma (RCC). IDH1 was observed lower expression in RCC cell lines. Phenotype experiment was carried out in the wild-type IDH1 and mutant IDH1^R132H^ plasmid treated cell line. The results showed that the wild-type IDH1 could significantly inhibit the proliferation, migration and promote the apoptosis of RCC cell lines, which were consistent with the IDH1's substrate α-KG. The mutant IDH1^R132H^ was found to lose this biological function of IDH1. Moreover, we verified the proliferation inhibition of IDH1 *in vivo*. In addition, we verified the correlation between IDH1 and hypoxia signal-related proteins *in vitro* and *in vivo*, specifically, IDH1 overexpression could significantly reduce the expression of HIF-1α and HIF-2α proteins and its downstream proteins (VEGF, TGF-α). Furthermore, we preliminarily verified the possibility of α-KG in the RCC's treatment by injecting α-KG into the xenograft model. α-KG significantly reduced tumor size and weight in tumor-bearing mice. This study provided a new therapeutic target and small molecule for the study of the treatment and mechanism of RCC.

## Introduction

Renal cell carcinoma (RCC) is a common urinary malignant tumor, accounting for about 2%-3% of adult malignant tumors. Currently, the incidence rate and the mortality rate were increasing in worldwide 1. The clear cell renal cell carcinoma (ccRCC) is the primary type of RCC, which represents 75%-80% of all RCCs 2. Some studies showed that patients with ccRCC had a poor prognosis. The 5-year survival rate of ccRCC patients is about 60%, which is lower than other types of RCC patients 3-5.

There are exact evidences that the von hippel-lindau (VHL) tumor suppressor gene is mutated or inactivated on chromosome 3p25 in more than 80% of ccRCC patients 6-7. Generally, VHL mediates the degradation of hypoxia inducible factor (HIF) through ubiquitination. Vascular endothelial growth factor (VEGF), transforming growth factor (TGF) and platelet-derived growth factor (PDGF) are important target genes downstream of HIF 8. In addition, mTOR pathway is found to be abnormally activated in renal carcinogenesis 9, and mTOR can also activate HIF 10. Thus, the abnormal accumulation of transcription factor HIF in renal cell carcinoma leads to overexpression of downstream target genes, such as VEGF and PDGF, which accelerates cell proliferation, promotes angiogenesis and tumor growth. This is one of the key molecular events in the occurrence of renal cell carcinoma. HIF signaling plays a crucial role in the formation and development of renal cell carcinoma. Successful targeting of HIF and its downstream signaling pathway is the key to the treatment of metastatic renal cell carcinoma.

Under normal oxygen (>8%-10%), the hydroxylation of HIF-alpha by intracellular hydroxylase provides a binding site for VHL protein, which is a substrate-binding protein of ubiquitin protein complex CRL2, thus mediating the hydroxylation of HIF-alpha by polyubiquitination and degradation through proteasome degradation pathway 11. Prolyl hydroxylase (PHD) is a common HIF-alpha hydroxylase. PHD-mediated hydroxylation occurs on two very conserved proline residues of HIF-alpha (Pro402 and Pro564 on HIF-1-alpha). The hydroxylation of any of these residues will provide a binding site for VHL protein, thus mediating the degradation of hydroxylated HIF-alpha by polyubiquitination 11. PHD is a dioxygenase dependent on alpha-ketoglutarate (α-KG) and ferrous ion (Fe2+). They first transfer one oxygen atom to the proline residue of HIF-alpha, and then react another oxygen atom with α-KG. Similarly to members of the prolyl hydroxylase family, factor-inhibiting HIF-1 (FIH1) is also a α-KG and Fe2+ dependent dioxygenase. The difference is that FIH1 catalyzes the hydroxylation of a conserved aspartic acid residue (Asn803) on HIF-1-alpha, which prevents the recruitment of co-activators P300 and CBP by HIF-1-alpha, and inhibits the transcriptional activity of HIF-alpha 12. Briefly, α-KG can synergize hydroxylation modification of HIF-alpha with dioxygenase PDH and FIH1 to bind to ubiquitinated junction protein VHL in cells, thus mediating the degradation of hydroxylated HIF-alpha by multiubiquitination, suggesting that α-KG plays an important role in regulating the protein level and activity of HIF-alpha in cells.

Isocitrate dehydrogenase (IDH) is a key rate-limiting enzyme in the tricarboxylic acid cycle. It catalyzes the oxidative decarboxylation of isocitrate to oxalosuccinic acid, an intermediate product, and then oxidizes decarboxylation to produce α-KG and carbon dioxide, providing energy and precursors for cell metabolism. IDH1 is one of three isomerases of IDH gene family which located in the cytoplasm and oxidase body and participates in the tricarboxylic acid cycle and provides energy. The products produced by IDH1 catalytic reaction include α-KG and reduced coenzyme II (NADPH). NADPH, as a donor of reductive hydrogen *in vivo*, participates in the oxidative stress response of cells on the one hand, and also participates in the oxidation process of unsaturated fatty acids 13-14.

Parsons *et al*. 15 have found that IDH1 had a high mutation rate in glioma, and the mutation only occurred in the fourth exon of IDH1 gene, causing 132 arginine to become histidine IDH1^R132H^. Mutant IDH1^R132H^ could lead to changes in enzyme activity and could not produce substrate molecule α-KG. Zhao et al. found that the mutated IDH1 was a dimer rather than a homodimer. The mutation results in a decrease in intracellular α-KG levels, and a series of reactions lead to an increase in the stability of HIF-1-alpha, which activates the HIF signaling pathway and may ultimately promote tumor growth 16.

In this study, we found that the expression of IDH1 in ccRCC was significantly lower than that in normal renal tissues adjacent to cancer. The decrease of IDH1 expression in ccRCC resulted in the decrease of intracellular α-KG production, and the up-regulation of IDH1 expression in ccRCC could inhibit the growth of ccRCC. The reduction of α-KG production in ccRCC cells resulted in the accumulation of HIF-alpha in ccRCC, promoting the growth and malignant transformation of tumors. It was proved that IDH1 and small molecule α-KG had inhibitory effect on ccRCC. This study is expected to further explore the pathogenesis of ccRCC and find new therapeutic targets.

## Materials and Methods

### Clinical specimens and cell lines

The tumor and paracancerous tissue samples (n = 39) in this study were obtained from radical nephrectomy patients at Zhongnan Hospital of Wuhan University. The clinical, and pathological data record of all patients were collected, and two pathologists were invited to confirm the histology diagnosis independently. The study using clinical information and surgical tissue specimens was approved by the Ethics Committee at Zhongnan Hospital of Wuhan University (approval number: 2015029, Related File 1). All patients provided the informed consent. The procedures in this study were done in accordance with the ethical standards of the institutional and/or national research committee.

RCC cell lines (A498, 786-O, 769-P) and 293T cell line were cultured in RPMI1640 medium (Gibco, China), ACHN cells were cultured in minimum essential medium (MEM, Gibco, China), Caki-1 cells were maintained in McCoy's 5 A Medium (Gibco, China) supplemented with 10% FBS. Human renal proximal tubular epithelial cell line (HK-2) was maintained in KSF medium with epidermal growth factor as well as bovine pituitary extract (Gibco, Carlsbad, CA, USA). All cell lines were purchased from the Stem Cell Bank, Chinese Academy of Sciences in Shanghai, China. These cell lines were grown at 5% CO2, 37 °C in a humidified incubator (Thermo Scientific).

### Bioinformatics analyses

In addition to the data from our center, we also accessed microarray expression profiles of The Cancer Genome Atlas (TCGA) to obtain the clinical and cytogenetic data of the external RCC patients. Database for Annotation, Visualization and Integrated Discovery (DAVID) Bioinformatics Resources 6.8 (https://david.ncifcrf.gov) can help reveal the biological processes and signal pathways that IDH1 get involved in RCC tumorigenesis.

### Transfections and stable cell lines selection

IDH1-siRNA (si-IDH1) and control-siRNA (NC) were all synthesized by GenePharma Gene Co Ltd. in Suzhou, China. IDH1-LV (LV-IDH1) was synthesized by Genechem Co Ltd. In Shanghai, China. The sense sequence of IDH1-siRNA was 5′-AAAUGAUUCGUGUCAUUUCAUCUCC-3′, the sense sequence of control-siRNA (NC)/control-shRNA (NC) was 5′-UUCUCCGAACGUGUCACGUTT-3′. IDH1 cDNA (1245 bp) was polymerase chain reaction (PCR) amplified from cDNA library of human RCC cell lines, then was cloned into p3 × FLAG-CMV-14 empty vector. IDH1 forward primer sense EcoRI 5′-GCGAATTCAATGTCCAAAAAAAT-CAGTGGCG-3′, IDH1 reverse primer sense BamHI 5′-CCGGGATCCAAGTTTGG-CCTGAGCTAGTTTG-3′. Reference to the manufacturer's protocol, cells were transfected with plasmids or siRNA oligonucleotides using Lipofectamine 3000 transfection reagent. To select the stable cell line, ACHN cells were infected with IDH1-LV and control-LV. 24 h later, 5 μg/ml puromycin (Sigma, USA) was added into cell culture medium to last 14 days. The antibiotic-resistant cells were selected successfully.

### RNA isolation, reverse transcription and quantitative real time PCR (qRT-PCR)

With the manufacturer's protocol, HiPure Total RNA Mini Kit (Cat. #R4111-03, Magen, China) was used to isolate total RNA from cells and RCC tissues. We performed the reverse transcription with ReverTra Ace qPCR RT Kit (Toyobo, China). RT-PCR was conducted with iQTM SYBR® Green Supermix (Bio-Rad, USA). Table 1 listed the primer sequences. Fold enrichment was calculated with the 2 - ΔΔCt method relative to GAPDH.

### Flow cytometry analysis for cell apoptosis

The transfected RCC cells were gathered, centrifugated and washed with cold PBS once. Follow the manufacturer's protocol, cells were collected and stained by annexin V-fluorescence isothiocyanate (FITC)/PI Apoptosis Detection Kit (BD biosciences, USA) according to the manufacturer's instructions protocol and analyzed by the flow cytometry analysis.

### Pre-treatment of α-KG

ACHN and 786O cell lines were first incubated in a 6-well plate, When the cells were adherent and the cell density reached 60%, α-KG (Cat. #S6237, Selleck Chemicals, USA) was used for treatment. After IC50 (50%inhibiting concentration) was calculated by cell proliferation assay, 0, 100, 300 μM was selected as the concentration gradient in subsequent experiments. We used this concentration gradient to incubate ccRCC lines for 48 hours and then collect the cells for subsequent experiments. α-KG used in all experiments was dissolved in 1% DMSO (Cat. #S7159, Selleck Chemicals, USA).

### Isolation of total protein from ccRCC cells, Western blot analysis

After harvesting the RCC cells, the RCC cells were washed by PBS for 3 times, then lysed cells with mixed solution that contained RIPA buffer, phosphatase inhibitor and protease inhibitor (Sigma-Aldrich, USA), for 30 min on ice. Then centrifuging the cell lysates at 13,000 × g for 15 min at 4 ℃, the supernatant liquid was then collected. Bradford protein assay (Bio-Rad, Germany) was used to measure the protein concentration of the supernatant liquid. Western blot analysis was performed after the total protein samples were separated with 7.5-15% SDS-PAGE. When HIF-1α and HIF-2α were detected by western blot analysis, the RCC cells were treated with cobalt chloride for 12 hours. The immunoreactive bands were visualized with an enhanced chemiluminescence kit (Bio-rad, USA), and then detected with Molecular Imager Chemi Doc XRS + Imaging system (Bio-rad, USA). Table 2 and supplementary Table S1 list the primary antibodies as well as secondary antibodies, respectively. It should be noted that the antibody of IDH1 was purchased from Proteintech (Cat. #12332-1-AP). The immunogen of IDH1 polyclonal antibody is C-term-296aa of IDH1 protein, which contains the 132th amino acid sites. So IDH1 antibody could not recognize the IDH1 R132H mutant in Western blot assay. For all that, the results did not affect the conclusion of this study.

### Xenograft mouse model

The BALB/c-nu mice (male, 3-weeks old) were obtained from Beijing Vital River Laboratory Animal Technology Co., Ltd. (Beijing, China). The xenograft mouse model related experiment was carried out in The Laboratory Animal facility of Zhongnan Hospital of Wuhan University. First, lentiviruses were used to construct stable transfected NC and sh-IDH1 ACHN cell lines, respectively. Then, 1 × 10^6^ cells were collected by counting and then dissolved in 100 μl PBS, and then injected subcutaneously into mice to construct the xenograft models. We then observed tumor volume every three days and recorded it until all the mice were sacrificed at 6 weeks.

### Statistical analyses

All the results of the study were repeated at least three times. Two-tailed student's t test was used to calculate the significance between the two sets of data. The SPSS 16.0 and R was used to perform all statistical analyses. GO and KEGG analysis were performed by “clusterpofiler” R package under the R version 3.6.1, and a p value < 0.05 was considered statistically significant.

## Results

### IDH1 was low expressed in ccRCC tissues and cell lines

**Figure 1:**
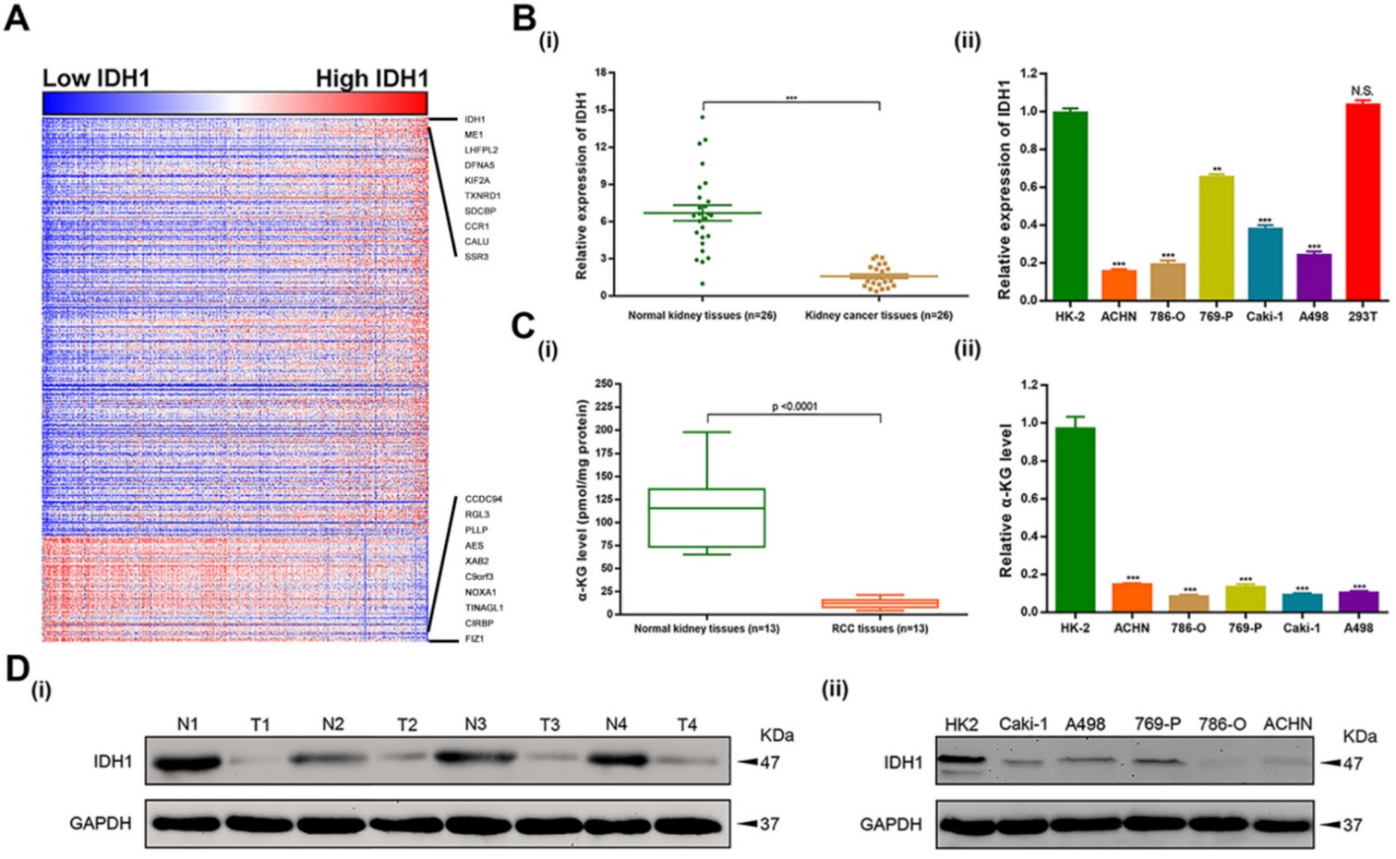
** Expression level distribution of IDH1 in RCC and cell lines. (A)** Heatmap of the 500 genes most relevant to IDH1 in TCGA-KIRC database, and the sequence of column samples is arranged according to IDH1 expression levels from low to high, red squares represent high expressions and blue squares represent low expressions. **(B-C)** Distribution of IDH1 transcriptional expression level and its substrate α-KG in (i) RCC tissues and (ii) cell lines. **(D)** Distribution of IDH1 protein levels in (i) RCC tissues and (ii) cell lines. *p < 0.05; **p < 0.01; ***p < 0.001; N.S. no significant.

To investigate the functional enrichment of IDH1 in ccRCC, relation analysis of IDH1 expression and top 500 correlation-genes in TCGA-KIRC database was performed. We identified 408 positive-related genes and 92 negative-related genes by the functional enrichment analyses (Figure 1A). qRT-PCR was used to analyze the transcription level of IDH1 in ccRCC (n = 26) and normal renal tissues (n = 26) as well as cell lines (HK-2, ACHN, 786-O, 769-P, Caki-1, A498, 293T). The results showed that IDH1 was lower expression in RCC tissues and cell lines (Figure 1B, 1D). The transcriptional level of metabolites of IDH1 (α-KG) was basically consistent with IDH1 (Figure 1C). Due to the low expression of ACHN and 786-O relative to HK-2 (P < 0.001, Figure 1B ii), two cell lines of ACHN and 786-O were selected for subsequent experiments.

### Functional annotation of IDH1 through correlation analysis

We downloaded the RNA-seq data (n = 607) of ccRCC of TCGA database from the UCSC Xena (*https://gdc.xenahubs.net/download/TCGA-KIRC.htseq_fpkm.tsv.gz*). We filtered out the low-signal genes, and selected the top 500 genes with the highest correlation for functional annotation analysis by calculating the Pearson correlation coefficient between all genes and IDH1. Through GO analysis, we found that IDH1 was related to biological processes such as Oxidation-reduction process, Protein phosphorylation, Metabolic process (Supplementary Figure S1A). The cellular component analysis showed that IDH1 mainly existed in cytoplasm and nucleus (Supplementary Figure S1B). Molecular function analysis exhibited that IDH1 mainly had protein binding, poly (A) RNA binding, identical protein binding, protein kinase activity and other molecular functions (Supplementary Figure S1C). KEGG analysis showed that IDH1 was mainly involved in metabolic, lysosome, protein processing in endoplasmic reticulum, carbon metabolism and other related signal pathways (Supplementary Figure S1D).

### Over expression and inactivation mutation of IDH1 repressed ccRCC cells proliferation and migration

**Figure 2:**
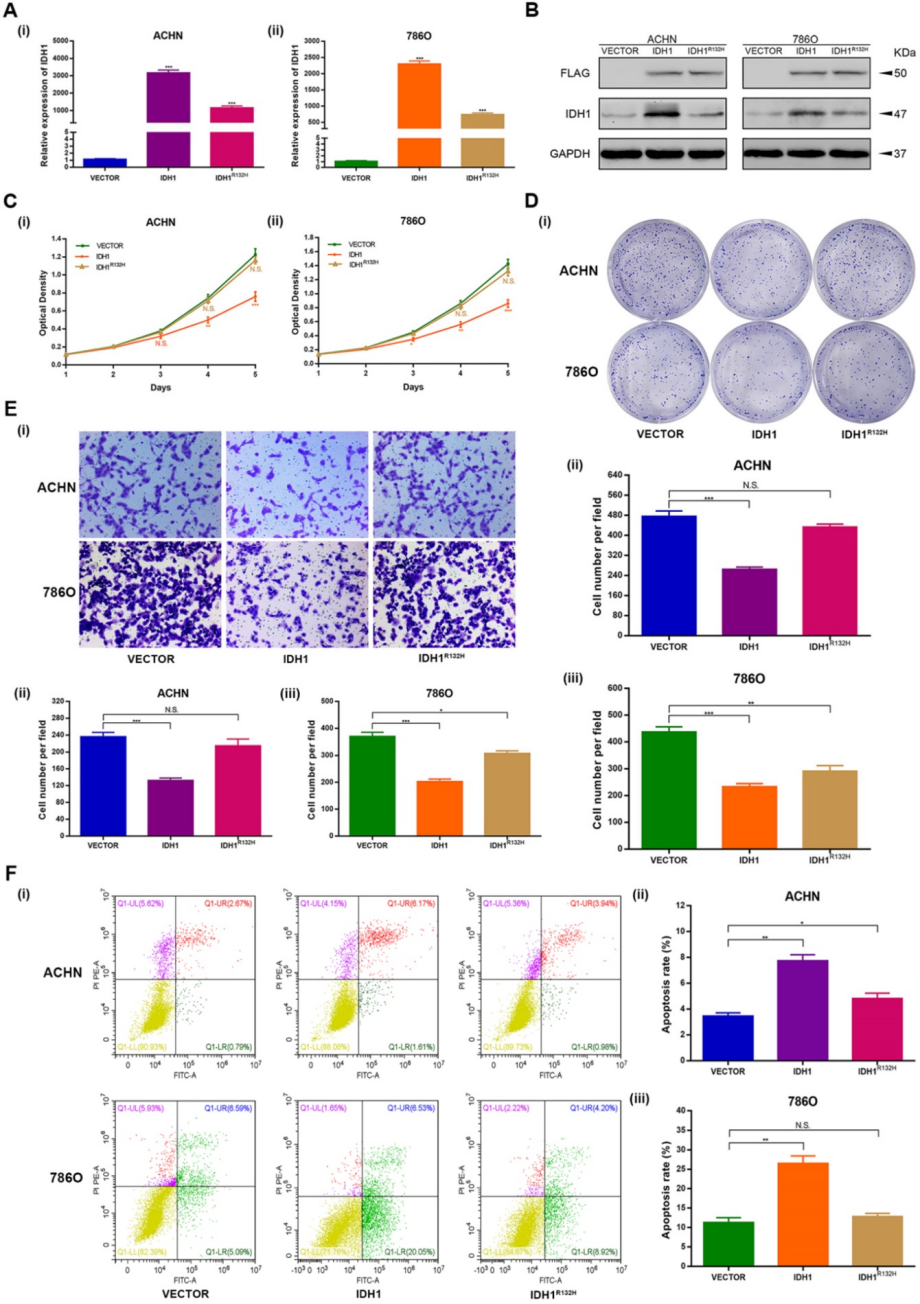
** Wild-type IDH1 inhibits proliferation and metastasis of RCC and promotes apoptosis. (A)** Transcriptional expression of wild-type IDH1 and mutant IDH1 was verified by qRT-PCR. **(B)** The translation level expression of wild-type IDH1 and mutant IDH1 was verified by WB assay. **(C)** MTT assay was used to determine the effect of wild-type IDH1 and mutant IDH1 on the proliferation ability of (i) ACHN and (ii) 786-O cell lines. **(D)** (i)The effect of wild-type IDH1 and mutant IDH1 on the proliferation ability of ACHN and 786-O cell lines was determined by clone formation assay. (ii-iii) The statistical histogram of the clone formation assay. **(E)** (i) Migration assay were conducted to measure the impact of wild-type and mutant IDH1 on migration capacity. (ii) The statistical histogram of the migration assay. **(F)** (i) The effect of wild-type and mutant IDH1 on cell apoptosis was measured by flow cytometry assay. (ii-iii) The statistical histogram of the cell apoptosis assay. *p < 0.05; **p < 0.01; ***p < 0.001; N.S. no significant.

The over expression and inactivation mutation function assays were performed to investigate the phenotypic changes of IDH1 in ACHN and 786-O cell lines. The p3xFlag-CMV-14-IDH1 were constructed and demonstrated good efficacy of IDH1 over expression at the transcription and translation levels as well (P < 0.001, Figure 2A-B). We modified the R132H mutation on the p3xFlag-CMV-14-IDH1 by molecular cloning technology. The results exhibited that the expression levels of transcription and translation of IDH1 ccRCC cell lines (ACHN, 786-O) transfected with IDH1^R132H^ mutant plasmid were lower than those of IDH1 wild-type ccRCC cell lines (Figure 2A-B).

MTT assays showed that the proliferation capacity of the ccRCC cell lines of IDH1 over expression group was significantly reduced, and there was no significant difference in the proliferation capacity of the cell lines transfected with IDH1^R132H^ plasmid (Figure 2C). The clone formation assays showed similar results, that is, the clone number and size of IDH1 overexpression treatment group were significantly smaller than that of the normal control group, and there was no significant difference between IDH1^R132H^ mutation group and normal control group (Figure 2D). The migration assay results displayed that the migration ability of ccRCC cell lines in IDH1 overexpressed group was reduced (P < 0.001, Figure 2E), and the migration ability of IDH1^R132H^ inactivated mutation treatment group was not significantly changed (Figure 2E).

### IDH1 over expression induced ccRCC cells apoptosis

Subsequently, we decided to explore whether IDH1 was involved in the regulation of apoptosis. Flow cytometry analysis showed that the apoptosis rate of the IDH1 over expression group was significantly increased compared with the normal control group (Figure 2F), and IDH1 inactivated mutation group was only significantly different from the normal control group in ACHN cell lines (P < 0.01, Figure 2F).

### IDH1 substrate α-KG is consistent with IDH1's biological function

**Figure 3:**
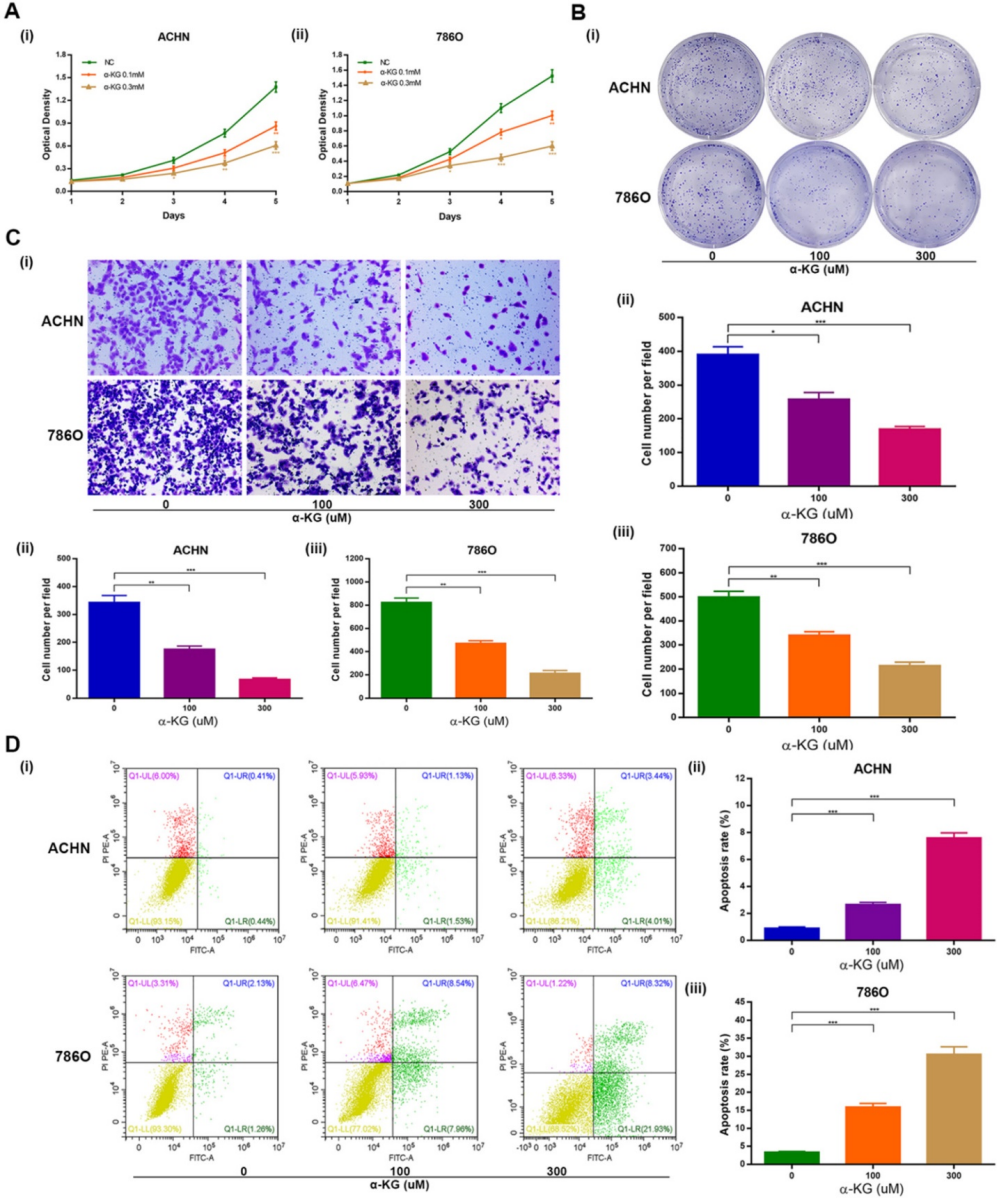
** Effect of IDH1 substrate α-KG on the biological function of RCC cell lines. (A)** The inhibition effect of α-KG on the proliferation of RCC lines was verified by MTT assay. **(B)** clonogenic forming assay showed that α-KG could significantly inhibit the proliferation of RCC cell lines **(C)** The migration assay verified the significant inhibition of α-KG on the migration ability of RCC cells. **(D)** α-KG significantly increased apoptosis in RCC cell lines. *p < 0.05; **p < 0.01; ***p < 0.001; N.S. no significant.

Then we studied the biological function of α-KG, the substrate of IDH1, and found that it was basically consistent with the biological function of IDH1. Specifically, MTT assays showed that α-KG with different concentration gradient (0, 100, 300 μM) could lead to decreased proliferation of ACHN and 786-O cell lines (Figure 3A). The clone formation assays showed that the higher the concentration of α-KG, the fewer clones and the smaller the size (Figure 3B). And through the migration assays, we found that α-KG can significantly inhibit the migration of ccRCC cell lines (P < 0.05, Figure 3C). The apoptosis assays results showed that α-KG significantly increased the apoptosis rate of ACHN and 786-O (P < 0.001, Figure 3D).

### IDH1 affects the RCC by down-regulating HIF-1α and HIF-2α

**Figure 4:**
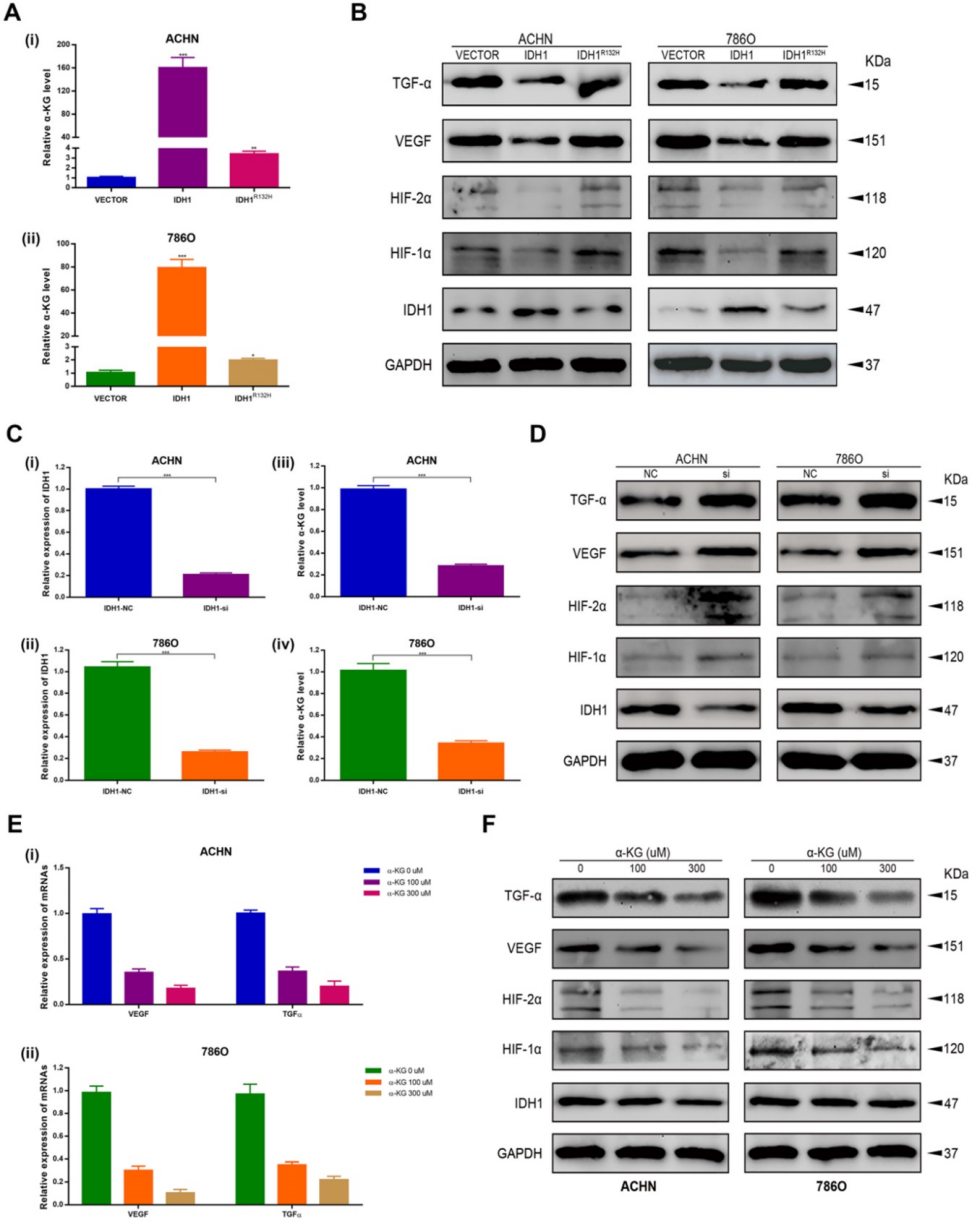
** IDH1 inhibits the development of RCC by repressing hypoxia-inducible factor 1. (A)** The effects of wild-type and mutant IDH1 plasmids on α-KG concentration were verified with a dedicated kit. **(B)** Western blot analysis of IDH1 affects representative proteins involved in the regulation of hypoxia signaling pathways: HIF-1α, HIF-2α, TGF-α and VEGF. **(C)** The knockdown efficiency of si-IDH1 was verified by qRT-PCR. **(D)** Compare the western blot results of hypoxia-related protein changes after IDH1 was knocked out. **(E)** qRT-PCR was used to detect the transcriptional changes of hypoxia-related proteins in α-KG after treatment. **(F)** WB was used to detect the translation level changes of hypoxia-related proteins in α-KG after treatment. *p < 0.05; **p < 0.01; ***p < 0.001; N.S. no significant.

We first verified the effect of IDH1 overexpressed plasmid and IDH1^R132H^ mutant plasmid on the level of IDH1 substrate α-KG (Figure 4A) and the knockdown efficiency of siRNA on ACHN and 786-O by qRT-PCR assays (Figure 4C). By western blot assay, we found that IDH1 overexpression group could reduce the protein levels of hypoxia signal-related pathway proteins (HIF-1α, HIF-2α, VEGF and TGF-α), and this change was not found in the IDH1^R132H^ mutant group (Figure 4B). We applied siRNA to renal cell lines and found that the results were the opposite of those in the overexpression group, that is, HIF-1α, HIF-2α, VEGF and TGF-α protein levels were significantly increased after IDH1 knockdown (Figure 4D). We also found similar results by using IDH1 substrate α-KG for renal cell lines (Figure 4E-F). In summary, our results showed that IDH1 inactivation mutation led to a decrease in substrate KG, which eventually lost its inhibition of HIF.

### Overexpression IDH1 suppressed RCC cell *in vivo*

**Figure 5:**
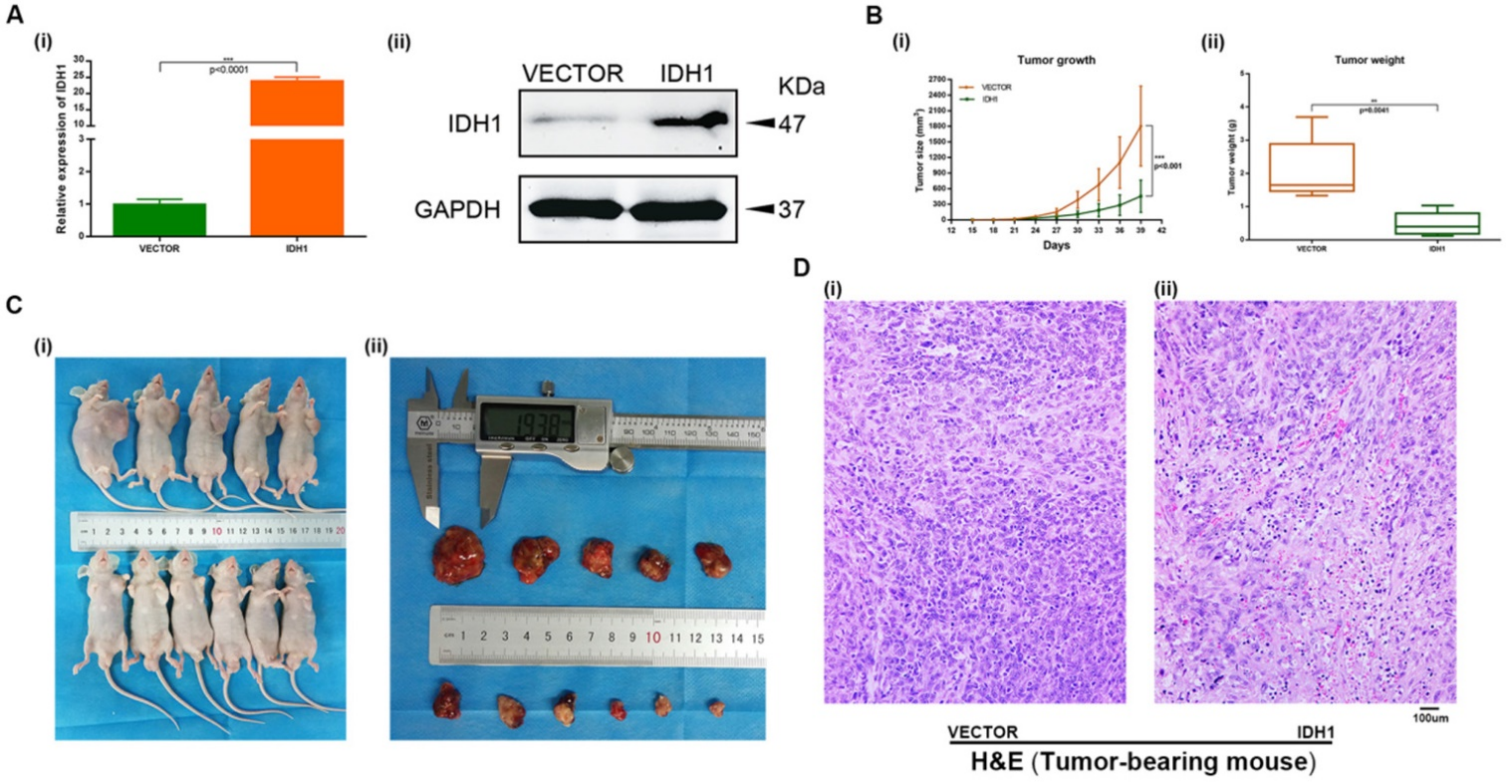
** Overexpression IDH1 suppressed RCC cell growth *in vivo*. (A)** Transcriptional and translational levels of overexpressed IDH1 lentivirus efficiency validation.** (B-C)** The continuous measurement of tumor growth activity and weighing mice body weight as well as dissected-tumor. **(D)** H&E Staining of tumor tissues in tumor-bearing mouse. *p < 0.05; **p < 0.01; ***p < 0.001; N.S. no significant.

**Figure 6:**
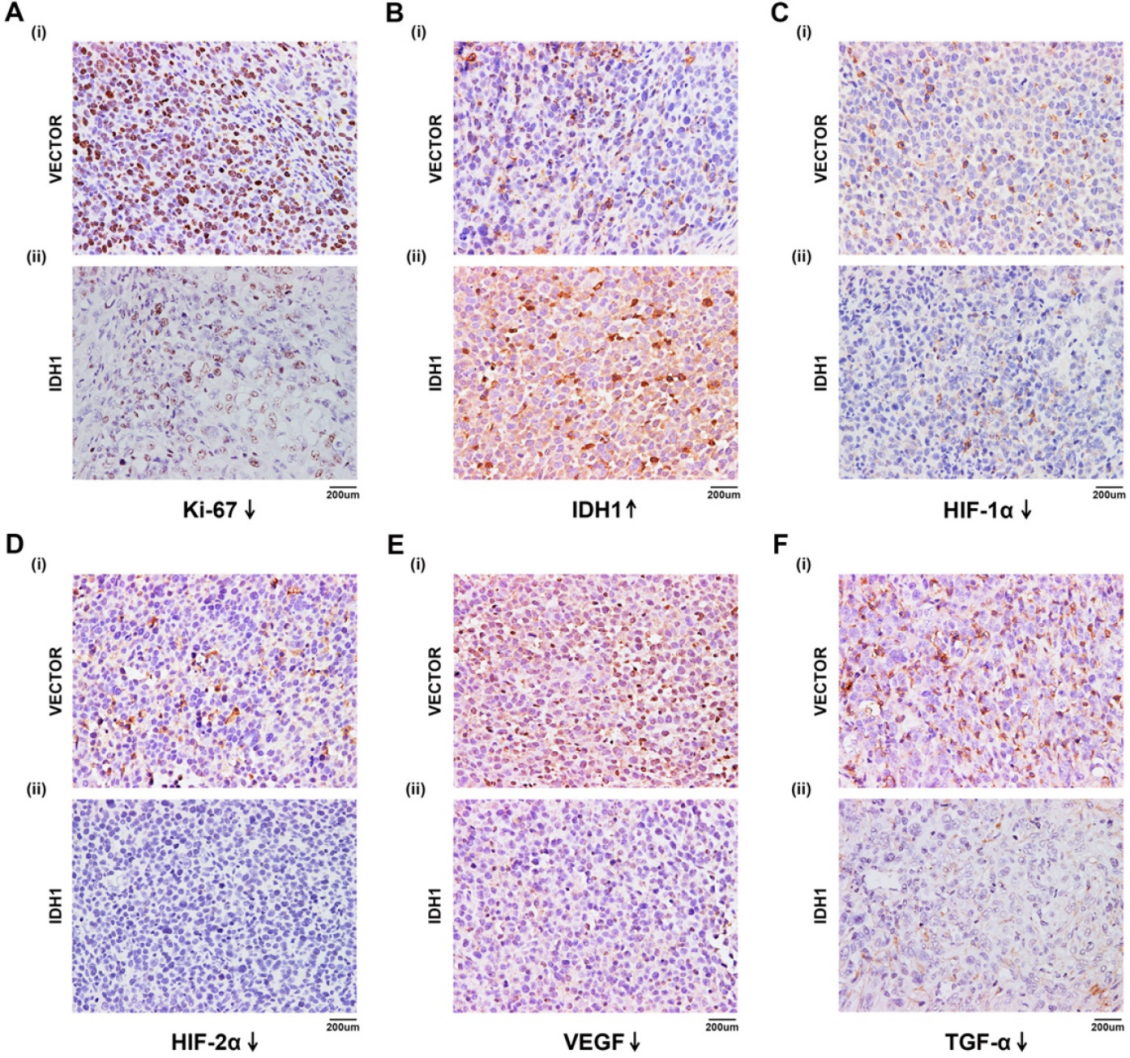
** IHC staining in tumor-bearing mouse. (A)** ki-67. **(B)** IDH1. **(C)** HIF-1α. **(D)** HIF-2α. **(E)** VEGF. **(F)** TGF-α.

A xenograft mouse model was established to study the effect of IDH1 and IDH1 substrate α-KG on RCC cell growth. We carried out xenograft mouse model treat with lentivirus IDH1. We first verified the overexpression efficiency of lentivirus IDH1 with qRT-PCR and WB assays (Figure 5A), and then plotted the tumor growth curve after tumor bearing and measuring the size and weight of mice, showing that overexpression of IDH1 could significantly inhibit tumor growth and significantly reduce tumor growth (Figure 5B-C). H&E staining of tumor of tumor-bearing mice showed that the number of tumor cells in tumors of IDH1 overexpressed mice decreased, suggesting that their tumor activity decreased (Figure 5D). IHC staining results showed that positive-staining of ki-67 decreased after IDH1 overexpression, suggesting that the tumor proliferation ability was reduced (Figure 6A). IHC staining verified the overexpression efficiency of IDH1, and the positive-staining of HIF-1α, HIF-2α, VEGF and TGF-α of the overexpressed IDH1 group were decreased (Figure 6B-F).

**Figure 7:**
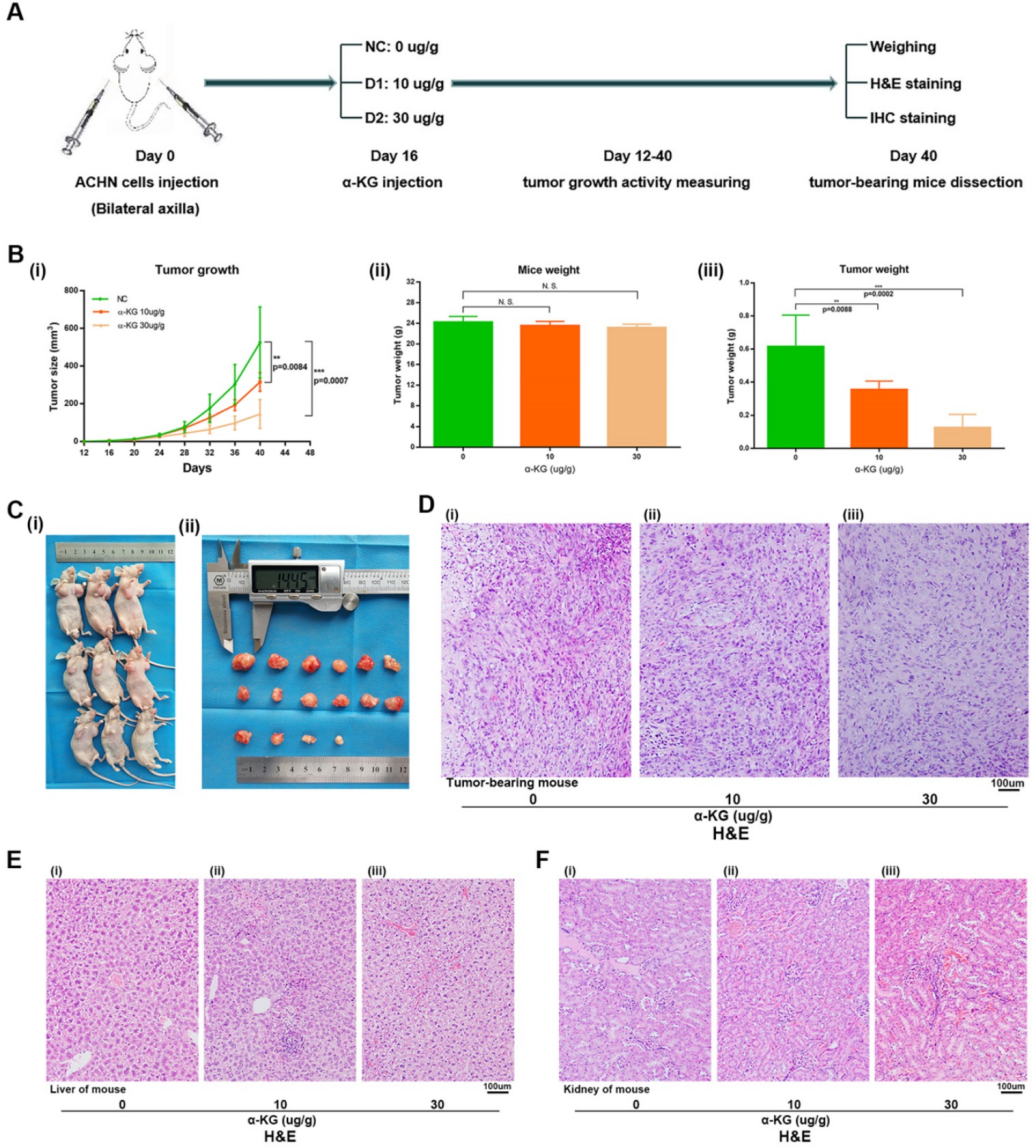
**α-KG suppressed RCC cell growth *in vivo*. (A)** Flow chart of xenograft mouse assay. **(B-C)** The continuous measurement of tumor growth activity and weighing dissected-tumor. **(D)** H&E Staining of tumor tissues in tumor-bearing mouse. **(E-F)** H&E staining of liver and kidney tissues in mice. *p < 0.05; **p < 0.01; ***p < 0.001; N.S. no significant.

For the α-KG-related experiments in xenograft mice, after 1 week of adaptive feeding, mice were subcutaneously injected with the same amount of ACHN cells, and α-KG was injected 16 days later. After 12-40 days, tumor size and weight of mice were measured, and experiments such as H&E staining and IHC staining were carried out (Figure 7A). Then the tumor size of mice treated with α-KG was measured, and the tumor growth rate (Figure 7B i, Figure 7C) and tumor weight of mice were significantly decreased compared with that of the NC group (Figure 7B iii). There was no significant difference in the total weight of mice in different groups (Figure 7B ii). H&E staining was performed on the tumor of tumor-bearing mice, and the results suggesting the tumor cells in the tumor of mice treated with α-KG decreased (Figure 7D). H&E staining of organs of xenograft mouse in each group showed no significant changes in liver and kidney of mice in the treatment group compared with those in the NC group (Figure 7E-F).

## Discussion

Several studies have confirmed the important role of IDH1 in tumors 17-22. Here, we confirmed the IDH1 in RCC suppressor role, and the hotspot mutations IDH1^R132H^ 23-25 will lose its inhibitory effect on RCC, we discussed the wild-type IDH1 underlying mechanisms of RCC inhibition, namely through regulating HIF-1α and HIF-2α to influence the occurrence and development of RCC, and the results *in vivo* and *in vitro* strict verification. In addition, we discussed the substrate of IDH1 α-KG potential as a treatment for RCC.

We first found that IDH1 expression in normal kidney tissues and cell lines was significantly higher than that in kidney cancer tissues and cell lines, and so was its substrate α-KG. It indicates that wild-type IDH1 has some inhibitory effect on renal carcinoma. Then we explored the biological function of wild-type IDH1 on RCC cells through MTT, migration and flow cytometry apoptosis assays, and the results showed that IDH1 could significantly inhibit the proliferation and migration of RCC cells and increase cell apoptosis. Moreover, we found that it's substrate α-KG also has a consistent biological function. In 92% of RCC, the VHL is mutated and inactivated 26, 27, losing its ability to regulate HIF-α and resulting in over accumulation of HIF-α. Excessive HIF-α would continue to up-regulate the expression of tumor-related genes 10, 28. So we studied the effect of wild-type IDH1 on hypoxia signal. By comparing the transcription level and translation level, we found that IDH1 could significantly inhibit the expression of HIF-α and its downstream proteins (VEGF, TGF-α, etc.). Besides, the inactivated mutation IDH1^R132H^ has a weaker effect on them than wild-type IDH1, indicating that IDH1 can influence the development of RCC through hypoxia signal, and IDH1^R132H^ mutation can weaken such influence. In addition, IDH1 plasmid and its substrate α-KG were used for *in vivo* experiments. We verified the inhibitory effect of IDH1 and its substrate α-KG on RCC by regularly measuring the tumor size and weight of tumor-bearing mice. Importantly, we verified the effect of IDH1 on HIF-related proteins through IHC in tumor-bearing mice. Finally, IDH1 substrate α-KG was used for *in vivo* experiments, and the results showed that α-KG also had a significant inhibitory effect on tumor size and weight in tumors bearing mice.

In summary, our study demonstrated that wild-type IDH1 can inhibit the proliferation, migration, and promote cell apoptosis of RCC. IDH1 was found to inhibit the development of RCC by inhibiting the expression of HIF and its downstream proteins VEGF and TGF-α. Moreover, IDH1's substrate α-KG has the same biological function, and we preliminarily verified the possibility of using α-KG as the treatment of RCC *in vivo* and *in vitro*.

## Conclusion

We proved through *in vitro* cell assay that wild-type IDH1 could inhibit the progression of RCC by inhibiting the proliferation and metastasis of RCC and promoting the apoptosis of RCC cell, and verified the inhibitory effect of IDH1 on RCC and the inhibitory effect of IDH1 substrate α-KG on tumor through *in vivo* experiments, preliminarily verified the possibility that α-KG could be used for the treatment of RCC.

## Supplementary Material

Supplementary figures and tables.

## Figures and Tables

**Table 1:** List of primers for qRT-PCR

Gene name	Symbol	Forward primer	Reverse primer	Annealing Temperature (°C)	Length (bp)
Glyceraldehyde-3-phosphate dehydrogenase	*GAPDH*	GAAGGTGAAGGTCGGAGTC	GAAGATGGTGATGGGATTTC	56	197
Isocitrate dehydrogenase 1	*IDH1*	TGTGGTAGAGATGCAAGGAGA	TTGGTGACTTGGTCGTTGGTG	62	147
Transforming growth factor alpha	*TGF-α*	AGGTCCGAAAACACTGTGAGT	AGCAAGCGGTTCTTCCCTTC	59	87
Vascular endothelial growth factor A	*VEGF*	AGGGCAGAATCATCACGAAGT	AGGGTCTCGATTGGATGGCA	61	75

**Table 2:** List of primary antibodies

Antigens	Species antibodies raised in	Dilution (WB)	Dilution (IHC)	Supplier
IDH1, human	Rabbit, monoclonal	1:1,000	1:50	Proteintech, China, Cat. #12332-1-AP
FLAG, human	Rabbit, monoclonal	1:1,000	-	Abcam, UK, Cat. #ab205606
GAPDH, human	Mouse, monoclonal	1:2,000	-	Santa Cruz Biotechnology Inc., USA, Cat. #sc-365062
Ki-67, human	Rabbit, monoclonal	-	2 ug/ml	Novus Biologicals, USA, Cat. #NBP2-19012
HIF-1 alpha, human	Rabbit, polyclonal	1:500	1:50	Novus Biologicals, USA, Cat. #NB100-105SS
HIF-2 alpha, human	Rabbit, polyclonal	1-2 ug/mL	1:100	Novus Biologicals, USA, Cat. #NB100-122SS
VEGF, human	Rabbit, monoclonal	1:2,000	1:250	Abcam, UK, Cat. #ab32152
TGF-α, human	Rabbit, monoclonal	1:1,000	1:4,000	Abcam, UK, Cat. #ab208156

## Abbreviations

RCC: renal cell carcinoma
FBS: fetal bovine serum
GEO: gene expression omnibus
GO: gene ontology
GSEA: gene set enrichment analysis
KEGG: kyoto encyclopedia of genes and genomes
PCR: polymerase chain reaction
qRT-PCR: quantitative real time polymerase chain reaction
α-KG: α-ketoglutarate
HIF-1α: hypoxia inducible factor 1α
HIF-2α: hypoxia inducible factor 2α
