# Supplementary material for: Wild-type IDH1 inhibits the tumor growth through degrading HIF-α in renal cell carcinoma
Source: Int J Biol Sci. 2021 Mar 25;17(5):1250–62. doi: 10.7150/ijbs.54401 (PMC8040470; doi:10.7150/ijbs.54401)
Supplement: Supplementary file 1 — Supplementary figures and tables. [file ijbsv17p1250s1.pdf]

Supplementary Figure S1. The functional annotation of IDH1 by GO and KEGG analyses. (A) Biological process. **(B)** Cellular component. **(C)** Molecular function. **(D)** KEGG.

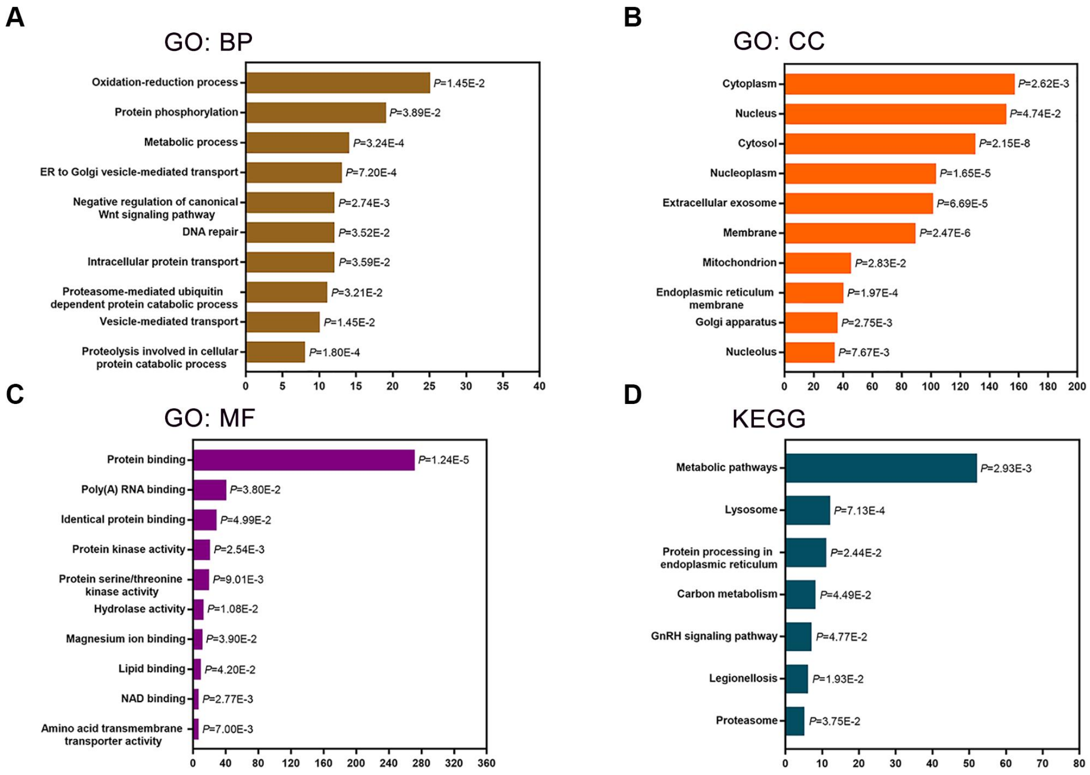

Supplementary Table S1. List of secondary antibodies and counterstaining of nuclei.

Secondary detection system used	Host	Method	Dilution	Supplier
Anti-Mouse-IgG (H+L)-HRP	Goat	WB	1:10,000	Sungene Biotech, China, Cat. #LK2003
Anti-Rabbit-IgG (H+L)-HRP	Goat	WB	1:10,000	Sungene Biotech, China, Cat. #LK2001
Anti-rabbit IgG (H+L), F(ab')2 Fragment (Alexa Fluor® 488 Conjugate)	Goat	WB	1:50	Cell Signaling Technology, USA, Cat. #4412
Anti-mouse IgG (H+L), F(ab')2 Fragment (Alexa Fluor® 555 Conjugate)	Goat	WB	1:50	Cell Signaling Technology, USA, Cat. #4408

Related File 1. Ethics Committee Approval (number: 2015029).

武汉大学中南医院医学伦理委员会科研项目伦理审批件

科伦[2015029]

项目名称	泌尿系疾病发病机制及分子诊治的科学研究				
申报单位	武汉大学中南医院				
项目负责人	王行环			科室	泌尿外科
课题来源	武汉市科技局				
审查文件	详见附件				
审查类别	初始审查	审查方式	会议审查		
审查日期	2015-07-29	审查地点	中南医院门诊 13 楼 2 号会议室		
出席人员	实到 10 人	回避 0 人		弃权 0 人	
投票结果	同意 3 票	作必要的修正后同意 7 票	作必要的修正后重审 0 票	不同意 0 票	暂停或终止已经批准的试验 0 票
审查意见	经本伦理委员会审查，审查决定如下：<br>☐同意 ■作必要的修正后同意 ☐作必要的修正后重审<br>☐不同意 ☐终止或暂停已批准的试验<br>主任委员或副主任委员签字：[illegible]<br>武汉大学中南医院医学伦理委员会（盖章）：<br>日期：2015-7.31				

注意事项：

1. 请遵循 CFDA/GCP 原则和《赫尔辛基宣言》、遵循本伦理委员会批准的方案开展临床研究，保护受试者的健康与权利。
2. 研究过程中，对研究方案和知情同意书等相关文件的修改，均须得到伦理委员会审查同意后方可实施。
3. 发生严重不良事件或影响研究风险受益比的非预期不良事件、违背方案、暂停/提前终止研究须及时报告本伦理委员会。
4. 根据项目来源类别提交跟踪审查材料。

伦理委员会声明：本伦理委员会严格按照中国 GCP 及相关法律法规组成及工作。

伦理委员会地址：湖北省武汉市武昌区东湖路 169 号；邮编 430071；电话：027-67812787。

复 审 表

项 目	泌尿系疾病发病机制及分子诊治的科学研究		
申办者			
方案版本号		方案版本日期	
知情同意书版本号		知情同意书版本日期	
研究专业/PI	王行环/泌尿外科		
审查意见：<br>1.所做修改是否符合上次审查意见<br>☑是 ☐否<br>2.是否需要进一步修正<br>☐是 ☑否<br>3.是否提交会议审查<br>☐是 ☑否			
主审委员审查意见	☑同意 ☐作必要的修正后同意 ☐作必要的修正后重审<br>☐不同意 ☐终止或暂停已批准的试验<br>建议：<br>主审委员签名：[illegible] 日期：2016.2.3		
医学伦理委员会评审结果：<br>☑同意 ☐作必要的修正后同意 ☐作必要的修正后重审<br>☐不同意 ☐终止或暂停已批准的试验			
主任委员或副主任委员签名/日期	[illegible] 2016.2.3		

Approval by the Ethics Committee at Zhongnan Hospital of Wuhan University for the microarray and RT-PCR analysis using RNA isolated from human bladder cancer tissues and normal bladder tissues from donors by accidental death.
